# Ferroptosis: A new therapeutic target for bladder cancer

**DOI:** 10.3389/fphar.2022.1043283

**Published:** 2022-11-03

**Authors:** Fan Zeng, Yunping Lan, Ning Wang, Xiaobo Huang, Qiao Zhou, Yi Wang

**Affiliations:** ^1^ Department of Critical Care Medicine, Sichuan Academy of Medical Science, Chengdu, China; ^2^ Department of Rheumatology and Immunology, Sichuan Academy of Medical Science and Sichuan Provincial People’s Hospital, University of Electronic Science and Technology of China, Chengdu, China; ^3^ Clinical Immunology Translational Medicine Key Laboratory of Sichuan Province, Sichuan Provincial People’s Hospital, University of Electronic Science and Technology of China, Chengdu, China

**Keywords:** bladder cancer, ferroptosis, iron metabolism, system Xc-GSH-GPX4 signaling pathway, metastasis, treatment

## Abstract

Bladder cancer (BC) is the most frequent type of urinary system cancer. The prognosis of BC is poor due to high metastasis rates and multidrug resistance. Hence, development of novel therapies targeting BC cell death is urgently needed. As a novel cell death type with strong antitumor potential, ferroptosis has been investigated by many groups for its potential in BC treatment. As an iron-dependent cell death process, ferroptosis is characterized by excessive oxidative phospholipids. The molecular mechanisms of ferroptosis include iron overload and the system Xc-GSH-GPX4 signaling pathway. A recent study revealed that ferroptosis is involved in the metastasis, treatment, and prognosis of BC. Herein, in this review, we comprehensively summarize the mechanism of ferroptosis, address newly identified targets involved in ferroptosis, and discuss the potential of new clinical therapies targeting ferroptosis in BC.

## 1 Introduction

As a genitourinary system tumor, bladder cancer (BC) is the most frequent type of urinary system cancer, accounting for over 570,000 new patients and 210,000 deaths globally in 2020 (Sung et al., 2021). According to the depth of invasion, bladder cancer can be subdivided into two categories: nonmuscle-invasive BC (NMIBC) and muscle-invasive BC (MIBC). BC patients have a comparatively high risk of mortality without proper treatment. In Europe, the standard relative five-year survival rate for BC patients is less than 60%, and the five-year survival rate decreases to 5.5% following metastasis (Richters et al., 2020; Witjes et al., 2021). Therefore, it is crucial to investigate novel cell death signaling pathways to reduce drug resistance and provide more therapeutic options.

**FIGURE 1:**
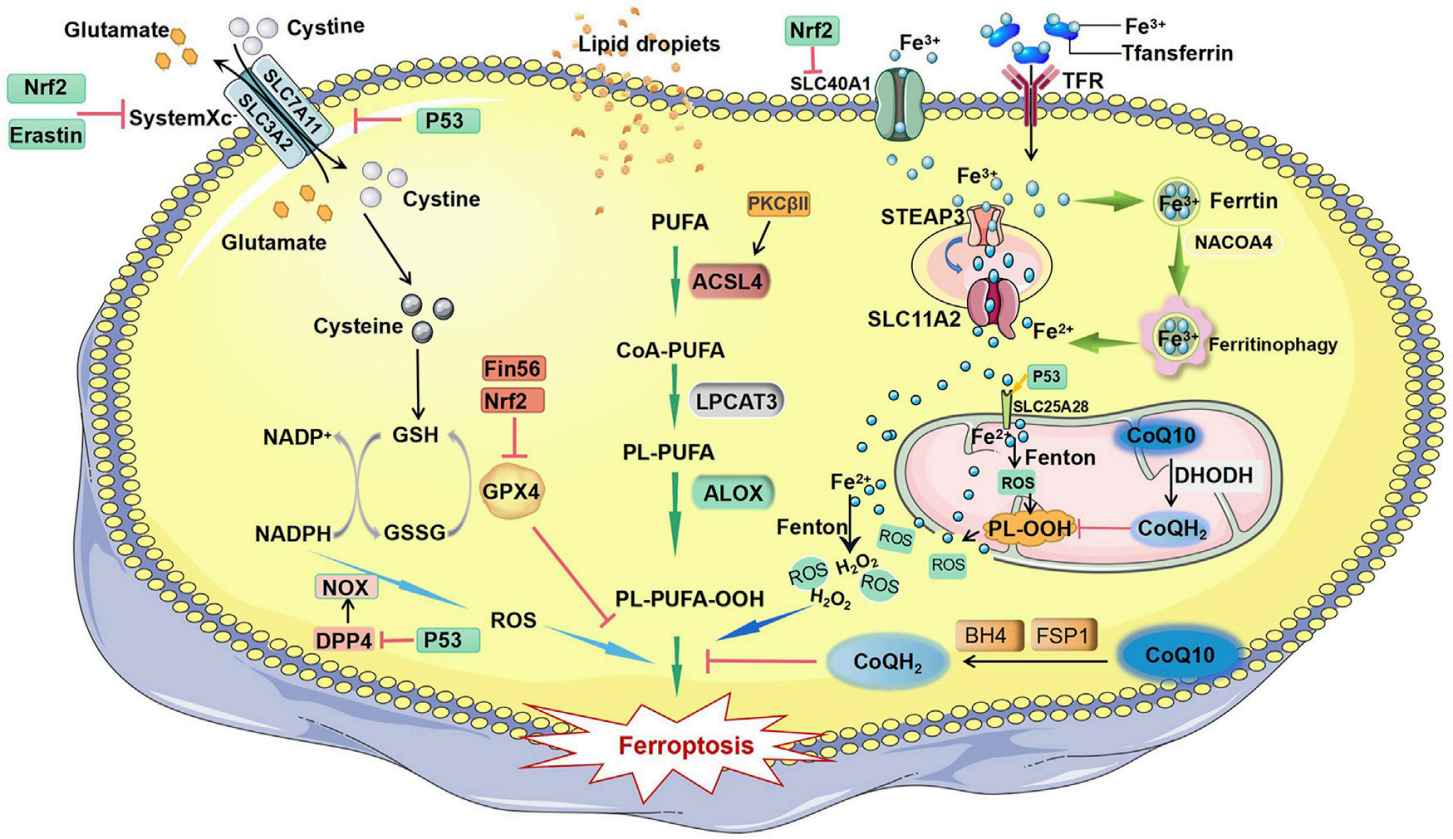
Mechanisms of ferroptosis. The initiation of ferroptosis requires two key signals, lipid peroxidation and accumulation of free iron. The generation of polyunsaturated phospholipids (by ACSL4 and LPCAT3) or PUFA-ePLs (by peroxisomal enzymes) and subsequent activation of ALOX have a major role in promoting lipid peroxidation, which can be inhibited by GPX4 and PKCβII. Extracellular iron enters the cell through TFR and facilitates the Fenton reaction to promote PUFA-PL oxidation. Some free iron stored in ferritin is released by ferritinophagy-mediated ferritin degradation. Iron can promote FSP1-CoQ10-NADPH pathways, and BH4 can block the propagation of phospholipid peroxidation and ferroptosis. Abbreviations: ALOX, lipoxygenase; ACSL4, acyl-CoA synthetase long chain family member 4; BH4, tetrahydrobiopterin; CoA-PUFA, coenzyme A-polyunsaturated fatty acid; CoQ10, coenzyme Q10; CoQH2, ubiquinol; DPP4, dipeptidyl peptidase 4; DHODH, dihydroorotate dehydrogenase; Fin56, ferroptosis inducer 56; FSP1, ferroptosis suppressor protein 1; GPX4, glutathione peroxidase 4; GSH, glutathione; GSSG, oxidized glutathione; H_2_O_2_, hydrogen peroxide; LPCAT3, lysophosphatidylcholine acyltransferase 3; Nrf2, nuclear factor erythroid 2-related factor 2; NADPH, nicotinamide adenine dinucleotide phosphate; NADP+, nicotinamide adenine dinucleotide phosphate; NACOA4, nuclear receptor coactivator 4; NOX, NADPH oxidase; PKCβII, protein kinase C beta type isoform; PUFA, polyunsaturated fatty acid; PL-PUFA, phospholipid-containing polyunsaturated fatty acid; PL-PUFA-OOH, phospholipid with a peroxidized polyunsaturated fatty acyl tail; P53, protein 53; PL-OOH, phospholipid hydroperoxide; ROS, reactive oxygen species; Slc7A11, solute carrier family 7 member 11; SLC11A2, solute carrier family 11 member 2; SLC25A28, solute carrier family 25 member 28; SLC40A1, solute carrier family 40 member 1; STEAP3, six transmembrane epithelial antigen of the prostate 3; TFR, transferrin receptor.

Ferroptosis is a type of regulated cell death that differs from necrosis or apoptosis. It is characterized by iron dependence. An imbalance in the generation and degradation of intracellular reactive oxygen species (ROS) results in reduced cellular antioxidant capacity, unrestricted lipid peroxidation, and plasma membrane rupture, finally causing ferroptosis (Aschner et al., 2022; Ozkan and Bakar-Ates, 2022). During ferroptosis, mitochondria are characterized by shrinking, absence of cristae, and increased membrane density. Compared with normal cells, tumor cells require more iron to support their proliferation. This means that tumor cells are more susceptible to iron-catalyzed necrosis. Therefore, ferroptosis has drawn increasing attention because it provides a promising avenue for oncotherapy (Bano et al., 2022; Bi et al., 2022). In the current review, we comprehensively summarize ferroptosis-related mechanisms (Figure 1), discuss newly identified targets involved in ferroptosis, and assess the potential of new clinical therapies targeting ferroptosis in BC.

## 2 Molecular mechanisms of ferroptosis

Lipid peroxidation and iron overload in cells are two key signals that induce ferroptosis. Among them, excessive lipid peroxidation is the core driving mechanism of ferroptosis. Polyunsaturated fatty acids (PUFAs) are important components in the formation of cell membranes (Kagan et al., 2017; Wenzel et al., 2017).

### 2.1 Lipid peroxidation in ferroptosis

PUFAs are components of the cell membrane and regulate many biological functions, including immunity, inflammation, and cellular growth, and also play a crucial role in promoting ferroptosis (Yin et al., 2011; Magtanong et al., 2019). The biosynthesis and transformation of PUFAs in cell membranes require acyl coenzyme A (CoA) synthetase long-chain family member 4 (ACSL4) and hemolytic phosphatidylcholine acyl transferase 3 (LPCAT3) enzymes (Kagan et al., 2017; Liu et al., 2022a). Under the catalysis of ACSL4, long-chain PUFAs (such as free arachidonic acid or epinephrine) combine with coenzyme A to form PUFA-CoA, which is then promoted by lysophosphatidylcholine acyl transferase 3 (LPCAT3). The esterification reaction produces the membrane phospholipid of polyunsaturated fatty acids (PUFA-PL). Exogenous administration of the monounsaturated fatty acid (MUFA) oleic acid (OA, C18:1) can compete with PUFAs for incorporation into phospholipids (PLs) and therefore inhibit erastin-induced ferroptosis (Yin et al., 2011). Because of the presence of bisallylmoieties in PUFAs, PUFA-PLs are prone to peroxidation to form PL-PUFA-OOH (Yagoda et al., 2007; Doll et al., 2017; Sung et al., 2021). After oxidation by lipoxygenases (ALOXs) or cytochrome P450 oxidoreductases (PORs), harmful lipid peroxidation products (phospholipid hydroperoxides, PLOOHs) are formed, causing rapid and irreparable damage to cell membranes and eventually leading to cell death (Li et al., 2021). Some studies have found that lipid peroxides can activate protein kinase βII (PKCβII) and that the activated PKCβII can phosphorylate the T328 site of ACSL4, promoting activation of ACSL4. The active ACSL4 promotes the synthesis of unsaturated fatty acid phospholipids and induces the production of lipid peroxides (Liu et al., 2021) (Liu et al., 2022a).

### 2.2 Iron overload in ferroptosis

Iron overload in cells is also an important cause of ferroptosis. The Fe^3+^ in the blood circulation is bound to transferrin (Tf). Subsequently, the transferrin receptor (TFRC) recognizes Fe^3+^ and transports it into the cytoplasm. Some of the iron is stored in ferritin, and the rest is reduced to divalent iron ions by an endosomal membrane protein, namely, STEAP3, and released into the cytoplasm through solute carrier family 11 member 2 (SLC11A2) (Gao et al., 2015; Li et al., 2021). When iron homeostasis is unbalanced in the body, nuclear receptor coactivator 4 (NCOA4) transports ferritin to lysosomes where it undergoes autophagic degradation, releasing free iron ions (Santana-Codina et al., 2021; Zhou et al., 2022). Fe^2+^ is unstable and easily oxidized and can interact with H_2_0_2_ produced by mitochondria. Hydroxyl radicals are generated through the Fenton reaction, which further causes PUFA-PL peroxidation and triggers ferroptosis (Hou et al., 2016; Zheng and Conrad, 2020). Increased levels of intracellular iron can enhance the sensitivity of cells to ferroptosis. Recent research has revealed that mitochondria play a crucial role in regulation of ferroptosis and are also the main source of reactive oxygen species (ROS) (Takashi et al., 2020). In the early process of erastin-induced ferroptosis, intracellular lipid oxygen species localize to mitochondria and subsequently appear in other cellular regions, such as the cell membrane. Mitochondria produce ROS and convert them to H_2_O_2_ *via* superoxide dismutase. H_2_O_2_ reacts with labile iron through the Fenton reaction to generate hydroxyl radicals, thereby promoting PUFA-PL peroxidation (Zheng and Conrad, 2020; Lei et al., 2022). In the absence of cysteine, glutamine changes the electron transport activity of protein complexes in the inner mitochondrial membrane by regulating the mitochondrial tricarboxylic acid cycle, causing the mitochondrial membrane potential to hyperpolarize, accelerating GSH depletion, promoting lipid ROS accumulation, and inducing ferroptosis (Gao et al., 2019).

### 2.3 System xc-GSH-GPX4 signaling pathway in ferroptosis

The system Xc-GSH-GPX4 pathway is the predominant signaling pathway for ferroptosis. System Xc belongs to the heterodimeric amino acid transporter family. It is a heterodimer that includes solute carrier family 3 member 2 (SLC3A2) and solute carrier family 7 member 11 (SLC7A11) linked through disulfide bonds. SLC3A2 and SLC7A11 are both embedded in the cell membrane. SLC7A11, the major functional subunit in system Xc-, is responsible for transportation of cystine into cells (Koppula et al., 2021). Cystine is reduced to cysteine after entering cells (Koppula et al., 2018; Liu et al., 2020). Cysteine is a key amino acid building block for glutathione (GSH), limiting the biosynthesis of reduced GSH (Meister, 1995). Glutathione peroxidase 4 (GPX4), which is also named phospholipid hydrogen peroxide glutathione peroxidase (PHGPx), is the major enzyme responsible for catalyzing the reduction of PLOOHS in mammalian cells (Ursini et al., 1982; Seibt et al., 2019). Through the catalytic selenocysteine residue of GPX4 and two electrons provided by glutathione (GSH), GPX4 can reduce phospholipids and cholesterol hydroperoxides to their corresponding alcohols. Erastin can prevent cystine import in a Gpx4 knockout mouse model, leading to PLOOH accumulation and causing unrepairable and rapid damage to membranes and ultimately cell death (Maiorino et al., 2018).

### 2.4 The role of p53 in ferroptosis

Gaurav et al. found that p53 is the most common genetic mutation in the population, with a mutation rate of 35% (Mendiratta et al., 2021). As a critical tumor suppressor gene, *p53* is located at the short arm of chromosome 17 and encodes the p53 protein. P53 can be induced by many stress signals, bind to DNA in the form of tetramers, and participate in cell cycle regulation, DNA repair, cell senescence, and death (Liu et al., 2019). The SLC7A11 gene is a known target of p53, and the activation of p53 leads to transcriptional repression of SLC7A11 (Jiang et al., 2015). P53 has also been reported to inhibit the uptake of cystine by downregulating SCL7A11 expression (Liu and Gu, 2022). This ultimately results in a decrease in the activity of glutathione peroxidase, a reduction in the antioxidant capacity of cells, and an enhancement of cell resistance to ferroptosis sensitivity (Liu and Gu, 2021; He et al., 2022; Hu et al., 2022). In an analysis of the expression levels of ferroptosis genes, GPX4, SLC7A11, and GSS were found to be highly expressed in some bladder cancer patients, and ACSL1 and ACSL4 were expressed at low levels. This means that bladder cancer cells can evade ferroptosis (Tang et al., 2022). The p53-SLC7A11 pathway has been reported to stimulate ferroptosis in a GSH-independent manner. Lipid oxidase ALOX12 is a key regulator of p53-dependent ferroptosis. ALOX12 is located on human chromosome 17p13.1 and is a popular site for heavy monoallelic deletions in human cancers. It has been reported that free ALOX12 oxidizes the polyunsaturated fatty acid chains of cell membrane phospholipids, leading to cell iron death. SLC7A11 can directly bind to free ALOX12 and limit its function. When p53 downregulates SLC7A11 expression, ALOX12 is released to induce ferroptosis (Chu et al., 2019). A p53 inducer can directly regulate the level of iPLA2β, but this response is very sensitive to the treatment time and concentration of the p53 inducer. The phospholipase iPLA2β can specifically hydrolyze the sn-2 acyl bonds of phospholipids, scavenge peroxidized phospholipids, and inhibit ROS-induced ferroptosis, independent of GPX4 and FSP1 (Li et al., 2021). P53 in mitochondria can bind with solute carrier family 25 member 28 (SLC25A28) to form a complex (p53-SLC25A28). Therefore, SLC25A28 activity is greatly enhanced, which leads to redox-active iron accumulation and ferroptosis (Zhang Z. et al., 2020). P53 can also directly bind to DPP4, preventing it from entering the cytoplasm and binding to NOX1, thereby promoting ferroptosis (Xie et al., 2017). In addition, the ferroptosis marker molecule PTGS2 was also confirmed to be a target gene of p53 (Yagoda et al., 2007; Doll et al., 2017; Guan et al., 2020; Sung et al., 2021). Numerous studies indicate that p53 promotes ferroptosis.

### 2.5 Other inhibitors of ferroptosis

Nuclear factor erythroid 2 (Nrf2), which plays a key role in antioxidant activity, is considered to be an important regulatory factor in ferroptosis. On one hand, Nrf2 can regulate the expression of GPX4 protein, while GPX4 overexpression results in ferroptosis resistance (Fan et al., 2017; Cui et al., 2021; Gao et al., 2022). On the other hand, Nrf2 can directly bind with the antioxidant-responsive element (ARE) sequence of the SLC7A11 subunit promoter, increasing the expression of SLC7A11 and increasing the GSH level to inhibit ferroptosis (Carpi-Santos and Calaza, 2018; Dong et al., 2020; Chen et al., 2022). Nrf2 regulates the intracellular free iron content by activating iron metabolism-related genes (SLC40A1). SLC40A1 can transport iron ions out of cells and inhibit the occurrence of ferroptosis (Chen et al., 2021) (Wu et al., 2017). The ferroptosis suppressor protein 1 (FSP1)-coenzyme Q10 (CoQ10) signaling pathway is another important ferroptosis inhibitory pathway. FSP1 is a flavoprotein oxidoreductase. FSP1 overexpression can suppress ferroptosis caused by GPX4 inhibition (Bersuker et al., 2019; Yan et al., 2021). Further studies found that FSP1 can act as an oxidoreductase to reduce COQ to COQ-H2 on the plasma membrane (Shukla and Dubey, 2018). CoQ, a lipophilic metabolite composed of a redox-active quinone head group and a long polyisoprenoid lipid tail, plays an essential role as a reversible redox carrier in the Golgi apparatus membrane and in plasma membrane electron transport. Its fully reduced form CoQH2 can act as a free-radical-trapping antioxidant to reduce lipid peroxidation free radicals (Stocker et al., 1991; Morre and Morre, 2011) (Gao et al., 2019) (Liu et al., 2022b). DHODH is the rate-limiting enzyme in the pyrimidine synthesis pathway and is an iron-containing flavin-dependent enzyme located on the inner mitochondrial membrane. One study found that a mitochondrial enzyme, dihydroorotate dehydrogenase (DHODH), on the outer surface of the inner mitochondrial membrane, can reduce coenzyme Q (CoQ) to ubiquinol (CoQH2) (Mao et al., 2021; Wang and Min, 2021). Tetrahydrobiopterin (BH4) is an alternative ferroptosis defense system independent of the GPX4-independent inhibitor of ferroptosis. BH4 is a powerful free radical-trapping antioxidant in cell membranes that promotes regeneration of CoQH2 and alpha-tocopherol to combat lipid peroxidation and ferroptosis (Crabtree et al., 2009; Kraft et al., 2020; Soula et al., 2020). BH4 is regenerated from its oxidized form boron dihydride (BH2) by dihydrofolate reductase (DHFR). Thus, DHFR inactivation significantly increases cellular susceptibility to ferroptosis (Hadian, 2020; Kraft et al., 2020).

## 3 The role of ferroptosis in bladder cancer

Abnormal expression of multiple ferroptosis-related proteins (FRPs), long noncoding RNAs (lncRNAs), microRNAs (miRNAs), and circular RNAs (circRNAs) has been found in bladder cancer specimens, indicating that ferroptosis plays an important role in the occurrence of BC (Zhang Y. et al., 2020). At the same time, FRPs and lncRNAs show good predictive value for prognosis and drug resistance in bladder cancer.

### 3.1 Predictive role of ferroptosis-related genes

Ferroptosis gene expression levels and the clinical data of BC patients were analyzed in The Cancer Genome Atlas (TCGA) and Gene Expression Omnibus (GEO) databases. It was found that a number of ferroptosis-related genes were abnormally expressed, such as SLC7A11, GPX4, TFRC, NCOA4, ACSL4, ALOX15, glucose-6-phosphate dehydrogenase (G6PD), and DPP4(66). Based on these ferroptosis regulator genes, various prognostic signatures have been established that can predict not only disease progression but also patient response to programmed death 1 (PD-1) and programmed death 1 (PD-L1) immunotherapy (Yi et al., 2021; Liu et al., 2022c; Gui et al., 2022). By analyzing data downloaded from multiple databases, Yue found that 23 FRGs were abnormally expressed and that low expression of recombinant heat shock 70 kDa protein 5 (HSPA5) and high expression of CDGSH iron sulfur domain 1 (CISD1) were associated with poor 1-, 3-, and 5-year overall survival (Yang et al., 2022). Xia used the Consensus Cluster Plus R package to divide the validated ferroptosis genes (VFGs) into four VFG clusters and found that there were differences in the prognosis of patients with different VGF clusters and tumor clinical manifestations. Scoring based on these phenotype-related genes revealed that VFR Cluster A had the highest score and was related to a worse response to PD-1 blockade immunotherapy (Xia et al., 2022).

### 3.2 Predictive role of lncRNAs

Accumulating evidence has demonstrated that multiple ferroptosis-related lncRNAs can be used to predict the prognosis and drug resistance of BC patients (Liang et al., 2021; Zhou et al., 2021; Li et al., 2022a; Liu et al., 2022a). LncRNAs, which are noncoding RNAs approximately 200 nucleotides in length, play a variety of roles in cancer immune responses and the tumor microenvironment (Yu et al., 2018; Yu et al., 2021). Liu found that lncRNAs were closely related to the tumor microenvironment and immunotherapy response. High-risk groups were associated with a poor prognosis and lower expression of certain proteins, including PD-1, PD-L1, and cytotoxic T lymphocyte-associated antigen-4 (CTLA-4), which indicate a poorer treatment response to immunotherapy (Liu et al., 2022a; Liu et al., 2022d). By analyzing ferroptosis-related lncRNA pairs and constructing a Cox proportional hazards model, it was found that multiple ferroptosis-related signaling pathways were altered in BC (Wang et al., 2022; Hou et al., 2022). The study showed high ferroptosis risk scores were correlated with high expression levels of the gene encoding PD-1, with a lower half-maximal inhibitory concentration (IC_50_) value for docetaxel, cisplatin, and pazopanib. This suggests that the combined use of ferroptosis-related drugs with immune checkpoint inhibitors in the high-risk group would benefit patients (Li et al., 2022b).

### 3.3 Potential therapeutic targets of circRNAs

CircRNAs are another type of noncoding endogenous RNAs, and they have circular configurations and stable structures (95). CircST6GAINAC6 was found to be significantly reduced in bladder cancer cells according to second-generation sequencing technology. One study showed that circST6GAINAC6 can bind to the N-terminus of heat shock protein 1, block phosphorylation at Ser15, activate the P38/MAPK pathway, and decrease the levels of SLC7A11 and GPX4, thereby promoting ferroptosis in bladder cells (96).

### 3.4 Potential therapeutic targets

Ferroptosis has a unique role in anticancer therapeutic strategies (Yi et al., 2021; Liu et al., 2022c; Gui et al., 2022). Treatments for induction of ferroptosis in bladder cancer are shown in Table 1. The system xc-GSH-GPX4 pathway is a classical inhibitory ferroptosis pathway. In recent years, most studies on potential therapeutic drugs or targets have focused on this pathway. SLC7A11 is an important functional component of system Xc-, and regulation of SLC7A11 can affect cell ferroptosis. Iron metabolism disorder is also an important cause of ferroptosis. One study found that RP11-89 mediates miR-129-5p expression and upregulates PROM2. Elevated PROM2 in cells is associated with diminished iron export, multivesicular body formation, and reduced mitochondrial abnormalities. Thus, RP11-89 may serve as a potential biomarker or therapeutic target in bladder cancer (Luo et al., 2021). The expression of ovarian tumor family deubiquitinase 1 (OUTB1) is significantly increased in human bladder cancer. Through *in vitro* and *in vivo* experiments, knocking out the OTUB1 gene was found to reduce the level of SLC7A11, inhibit the uptake of cystine by cells, and promote ferroptosis (Shimada et al., 2016). Epithelial membrane protein 1 (EMP1) was found to be downregulated in BC cells. In cells lacking EMP1, the addition of the ferroptosis inducer erastin promoted anti-ferroptosis cell death through upregulation of SLC7A11 expression (Hao et al., 2022). Thus, OUTB1 and EMP1 may be potential therapeutic targets.

**TABLE 1:** Treatments for induction of ferroptosis in bladder cancer.

Drug	Ferroptosis (inducer inhibitor)	Target gene	Mechanism	Model	References
7j	Inducer	GPX4	Inactivated GPX4, in lipid peroxidation ↑	*In vitro*	Chen et al. (2020)
OTUB1	Inhibitor	SLC7A11	SLC7A11↑, cystine ↑,GSH ↑,GPX4↑	*In vitro*/*in vivo*	Shimada et al. (2016)
Emp1	Inhibitor	SLC7A11	SLC7A11↑, cystine ↑,GSH ↑,GPX4↑	*In vitro*	Hao et al. (2022)
Erianin	Inducer	NRF2	Inactivated NRF2, ROS↑, GSH↓	*In vitro*/*in vivo*	Xiang et al. (2021)
CircST6GAINAC6	Inducer	SLC7A11	SLC7A11↓, GPX4↓, activate the P38/MAPK pathway	*In vitro*	96
Fin56	Inducer	GPX4	GPX4↓, lipid peroxidation ↑	*In vitro*	97
LncRNA RP11-89	Inhibitor	PROM2	Fe^3+^↓, Ferritin↓, SLC7A11↑, GPX4↑	*In vitro*	Luo et al. (2021)
Au@Chl/Fe	Inducer	Iron	ROS↑, GSH↓, lipid peroxidation ↑ in PDT	*In vitro*/*in vivo*	Liao et al. (2022)
Fe3O4@Chl/Fe CNPs	Inducer	Iron	Iron ion overload, ROS ↑,GSH↓, GPX4↓	*In vitro*/*in vivo*	Chin et al. (2022)
AuNRs&IONs@Gel	Inducer	Iron	Release iron ions in cell, ROS ↑	*In vitro*/*in vivo*	Guo et al. (2020)
Bupivacaine	Inducer	Fe^2+^	Fe^2+^↑, ROS↑, GPX4↓, MDA↑	*In vitro*/*in vivo*	Hao et al. (2022)
Huang qin	Inducer	FTH1	FTH1↓, Fe^2+^↑, ROS↑	*In vitro*/*in vivo*	Kong et al. (2021)
Gold clusters (PAA4, PAA5)	Inducer	GSH	GSH↓, GPX4↓	*In vitro*	Xiao et al. (2022)

Note. ↓stands for decreases the expression of, and ↑ stands for increases the expression of.

Abbreviations: Au@Chl/F, aurum@iron chlorophyll; AuNRs&IONs@Gel, aurum nanorods, and iron oxide nanoparticles@ gel; CircST6GAINAC6, circular RNA ST6GAINAC6; Emp1, epithelial membrane protein 1; Fe, ferrum; Fe_3_O_4_@Chl/Fe CNPs, ferroferric oxide@ iron chlorophyll cluster-structured nanoparticles; Fin56, ferroptosis inducer 56; FTH1, ferritin heavy chain; GSH, glutathione; GPX4, glutathione peroxidase 4; LncRNA RP11-89, long non-coding RNA RP11-89; MDA, malonaldehyde; NRF2, nuclear factor erythroid 2-related factor 2; OTUB1, ovarian tumor family deubiquitinase 1; PDT, photodynamic therapy; PROM2, prominin 2; ROS, reactive oxygen species; SLC7A11, solute carrier family 7 member 11.

## 4 Potential therapeutic drugs

### 4.1 Chemotherapy and immunosuppressants

For traditional chemotherapy, cisplatin, gemcitabine, or carboplatin have certain effects in the treatment of bladder cancer (Taber et al., 2020; Pfister et al., 2022). Other chemotherapies, such as quinazolinyl-arylurea derivative 7j, can bind to active GPX4, inhibit the sxc-/GPX4/ROS pathways, and induce ferroptosis (Chen et al., 2020). Immune checkpoint drugs, such as atezolizumab and pembrolizumab, are also effective against several diseases, including bladder cancer (Nadal and Bellmunt, 2019). Although single-drug treatment with immune checkpoint inhibitors (ICIs) has been successful, the long-term persistent remission rate is still very low, and many patients relapse. Early efforts to combine ICIs with traditional chemotherapy have shown a minimal impact on clinical benefits (Nadal and Bellmunt, 2019).

### 4.2 Traditional Chinese medicine

Traditional Chinese medicine has been used in the treatment of bladder cancer. Huang qin, which is extracted from the roots of *S. baicalensis Georgi* (Scutellariae Radix), triggers ferroptosis *in vitro* and *in vivo* by increasing intracellular chelate iron enrichment and ROS accumulation through overexpression of ferritin heavy chain 1 (FTH-1) (Kong et al., 2021). Erianin, a ferroptosis inducer, can promote ROS accumulation by inactivating nuclear factor E2-related factor (Nrf2) and GSH, thereby inducing ferroptosis in tumor cells (Xiang et al., 2021).

### 4.3 Nano drugs and gold clusters

A variety of nano drugs have been reported to specifically adhere to bladder cancer cells and deliver iron through endocytosis when exposed to laser irradiation, resulting in ROS generation and accumulation and subsequent GSH and GPX4 depletion, ultimately leading to ferroptosis (Liao et al., 2022). Another approach is to enhance photodynamic therapy (PDT)-chemodynamic therapy (CDT) sensitivity and induce toxicity (Chin et al., 2022; Liao et al., 2022). AuNRs&IONs@Gel is a gel delivery platform with embedded gold nanorods (AuNRs) and iron oxide nanoparticles (IONs). The IONs can be absorbed by bladder tumor cells and activate iron-mediated lipid peroxidation (Guo et al., 2020). The innovative antitumor mechanism of gold complexes has attracted attention. Gold clusters (PAA4 and PAA5) can trigger the rapid release of Au(l) ions from GSH, thus inhibiting thioredoxin reductases, stimulating oxidative reactions, and accelerating ferroptosis (Xiao et al., 2022).

### 4.4 Other potential therapeutic drugs

Many additional drugs have shown therapeutic prospects. The compound Fin56, a ferroptosis inducer derived from caspase-3/7-independent lethal 56, can induce ferroptosis by inhibiting mTOR-mediated autophagy to degrade GPX4 in BC cells (Shimada et al., 2016; Sun et al., 2021). Bupivacaine (0.25–16 mM), a common local anesthetic, was found to inhibit PI3K/AKT signaling pathway activity by increasing Fe^2+^ levels and ROS levels. Furthermore, it reduced GSH levels and increased malonaldehyde (MDA) levels in BC cells, which suppressed the growth of xenografted tumors and induced ferroptosis (Hao et al., 2022).

## 5 Conclusion and perspectives

Bladder cancer has a high recurrence rate and high morbidity and mortality. Current treatments cannot effectively cope with the resistance of cancer cells to existing chemotherapy drugs. With the development of precision medicine, precise disease diagnosis and individualized selection of the best treatment strategy have become a trend in developing treatment strategies. Ferroptosis is a new type of cell death that differs from apoptosis and necrosis. Many studies have demonstrated that ferroptosis is involved in the metastasis, treatment, and prognosis of bladder cancer. The prediction of prognosis and drug resistance based on a ferroptosis-associated gene-based molecular typing model has also received extensive attention. By analyzing the related ferroptosis gene data of bladder cancer patients in The Cancer Genome Atlas (TCGA) and Gene Expression Omnibus (GEO) databases, the constructed risk score model can predict bladder cancer prognosis and sensitivity to chemotherapy drugs. Therefore, based on the precise identification of biomarkers of ferroptosis in bladder cancer patients, it is expected that subsequent molecular targeted precision therapy can be achieved. Some conventional drugs that activate ferroptosis have shown favorable antitumor effects *in vivo* and *in vitro*. Through intravesical injection, drug-containing compounds can reach a high concentration in the bladder without entering the bloodstream, which avoids many side effects of systemic medication. At present, a variety of iron-containing nanomedicines have achieved good efficacy *in vivo*. This will be a new research direction. However, no drug targeting the canonical ferroptosis pathway has undergone clinical trials in bladder cancer. We believe that with more in-depth studies on the mechanisms and targets of action of ferroptosis inhibitors, clinical trials are essential. Clinical trials may become the key for future research. As shown here, targeting ferroptosis in bladder cells could become a new anticancer therapy approach in the future.
